# Knowledge of assessment about Tourette’s Syndrome among Medical Students and General Physicians in Jordan: A Cross-Sectional Study

**DOI:** 10.1192/j.eurpsy.2026.11540

**Published:** 2026-07-31

**Authors:** A. A. Alqudah, A. E. K. El-Lababneh, O. M. Bani Ahmad, S. B. Almasri, Khaled M. Al-Sawalmeh

**Affiliations:** Faculty of Medicine, Yarmouk University, Irbid, Jordan

## Abstract

**Introduction:**

Tourette’s syndrome (TS), a chronic, usually disabling neuropsychiatric disorder characterized by motor and vocal tics, patients are frequently misdiagnosed, or diagnosed lately. Patients are also present with additional psychological disorders like anxiety, depression, and ADHD. There is a large gap in research about Tourette’s syndrome (TS) in the Middle East as we noticed from our daily medical conversations in the medical field and social media, that most of the students and General Physicians have less information about what Tourette’s syndrome is and how to diagnose it.

**Objectives:**

The purpose of this study was to evaluate the general practitioners’ (GPs’) and medical students’ (Jordan’s) knowledge and understanding of TS.

**Methods:**

From December 2024 to May 2025, 387 participants—283 medical students and 104 general practitioners—from six public Universities and healthcare facilities in Jordan participated in a cross-sectional study. Knowledge in the areas of symptoms, causation, diagnosis, treatment, and misconceptions was evaluated using a validated 36-item questionnaire. Descriptive statistics, logistic regression chi-square, t-test, ANOVA, and correlation were used to examine the data using the (SPSS v26),.

**Results:**

Social media was the main source of knowledge for 53% of participants, and the majority (82.9%) had heard of TS. The greatest percentage of people knew about symptoms (73.9%), yet misconceptions about “mortality” (3.1%) remained. Every participant received a score of less than 50%, which is considered “poor knowledge.” The mean knowledge scores of females were higher than those of males (15.95 vs. 13.11, p = 0.018). Knowledge and GPA did not correlate (p = 0.414). Academic year and knowledge had a positive correlation (r = 0.357, p < 0.001). While gender and GPA were not significant predictors of awareness, academic year (OR 24.6–60.8, p < 0.01) and university affiliation were, according to logistic regression.

**Conclusions:**

The current study’s findings reveals that there is a significant lack of awareness and knowledge among both medical students in all public universities and generalphysicians in Jordan. Curricular reforms and training initiatives are needed.

**Disclosure of Interest:**

None Declared

